# National Immunization Therapeutic Advisory Group: it is time for experience sharing and best practice learning

**DOI:** 10.3402/jmahp.v3.29276

**Published:** 2015-09-14

**Authors:** Walter Ricciardi, Mondher Toumi

**Affiliations:** 1 European Public Health Association and, Department of Public Health, Catholic University of the Sacred Heart, Rome, Italy; 2 Faculty of Medicine – Public Health, University Aix-Marseille, Marseille, France

Vaccines bring multiple and various benefits to society. Vaccination has made a fundamental contribution to the decreased incidence of numerous infectious diseases and associated mortality. In developed countries, routine vaccination has led to complete eradication or control of several infectious diseases (1). Vaccination can control the transmission of the causative agent and limit associated infection outbreaks (2). The society as a whole benefits from vaccination, as vaccines exert their effect in both direct and indirect ways (herd effect). Thanks to the herd effect, unvaccinated individuals are also protected by reduction of population susceptible to infection (3). The value of indirect protection should be especially appreciated in the context of families’ dynamics and caregivers’ absenteeism. For example, children are primary vectors of some disease transmission. Vaccination may reduce spreading of the disease to parents (4). The same may be said about working grandparents who often take care of their grandchildren and who, due to age, can be even more susceptible to infection than parents (5, 6). The herd effect enables protection for the subpopulations that cannot benefit from direct protection, such as newborn infants, pregnant women, and immunocompromised individuals.

Apart from preventing transmission to parents and grandparents, vaccination prevents absenteeism and loss of productivity caused by the need for care of infected children. It has been demonstrated that varicella disease, rotavirus gastroenteritis, or influenza in children incurs considerable indirect costs as a result of parental absenteeism and loss of productivity (4, 7–11).

Prevention is the cornerstone of health enhancement and can be considered the first level of healthcare (12). Vaccination is broadly recognized as one of the primary prevention measures that promotes public health. Vaccination is often considered as one of the most cost-effective public health interventions (2, 13). Public health benefits provided by vaccines have been proved no matter what indicators were used, that is, avoided deaths, life-years saved, disability-adjusted life-years avoided, or quality adjusted life-years gained (14).

Vaccination can potentially improve population health contributing to economic growth. A healthy population generates higher incomes. This is achieved by a number of mechanisms: healthy children are able to acquire education more effectively, healthy adults are more productive and able to work longer, a healthy society tends to save more and to attract more foreign investment contributing to capital accumulation, job creation, and technological progress (15).

Vaccination can add to the sustainability of healthcare systems by avoiding unnecessary use of financial and human resources and freeing resources for other medical interventions. In the late 1990s, new vaccines, such as rotavirus, meningococcal, pneumococcal conjugate, and varicella vaccines came onto the market and have been shown to reduce the costs associated with hospitalizations and outpatient visits (16–22). In addition, rotavirus vaccine plays a role in prevention of nosocomial infections (23). By decreasing the incidence of diseases, vaccination also leads to a reduction in the consumption of medications (4). In the elderly, reduced medicines consumption is likely not only to reduce directly associated costs but also to prevent costs connected with side effects of medicines (24, 25). Vaccines contribute to cancer prevention. Nearly 20% of all cancers are associated with infectious agents. Among the most important causative agents are human papillomaviruses associated with most cervical and anal cancers as well as a fraction of oral cancers; hepatitis B virus and hepatitis C virus which can cause liver cancer; and *Helicobacter pylori* which is considered as one of the etiologic factors of stomach cancer (26). Vaccines are the most effective way of preventing some of these infections.

In spite of the fact that vaccination constitutes one of the most significant public health advancements, the time to effective populations’ access to new vaccines is heterogeneous and lengthy in developed countries, with an average of 6.4 years between European Marketing Authorization and effective populations’ access to new vaccines. The main driver of the delay in access is the time taken by the National Immunization Technical Advisory Groups (NITAGs) to issue vaccination recommendations guiding the executive policy decisions (27). Ricciardi et al. have reported on the disparities and shortages in NITAGs’ processes in 13 developed countries (Australia, Belgium, Canada, France, Germany, Hungary, Italy, the Netherlands, Spain, Sweden, Switzerland, the United Kingdom, and the United States) (28):

- In the majority of the 13 studied countries, information on NITAG's processes and policies was limited. The most restricted access to information on NITAG was observed in Italy, where there is not even an official website. The broadest scope of information was provided by NITAGs in United Kingdom, Germany, and the United States.
- Terms of reference, defining the official mandate of the NITAG, were available only for around half of the countries. Terms of reference did not cover all actual roles of NITAGs. Apart from the main mission assigned to all NITAGs (to provide advice for National Immunization Plan), other functions varied widely from one country to the other.
- NITAGs’ recommendations ultimately influenced policy decision making.
- Most NITAGs consisted of core members and stakeholders. The number of members ranged from 12 in Hungary to up to 48 in the United States. Too high a number of members may distract an efficient decision process (29). Apart from the United States and France, the number and function of members were not clearly defined. The profile of members varied greatly among countries.
- A declaration of conflict of interest was common practice among NITAGs, whereas remuneration of the members was only given in four countries. Lack of remuneration may lead members to give NITAG a low priority (29).
- The decision analysis frameworks were available for a limited number of NITAGs with only two countries (Germany and the United States) using a detailed and standardized methodology (the Grades of Recommendation, Assessment, Development and Evaluation – GRADE). Advice was publicly available in all selected countries except Italy, but access to meeting minutes or open meetings was much more limited.

Public trust in vaccination programs recommended by health authorities is crucial. To maintain trust and strengthen the reliability of immunization policies and programs, the transparency of NITAGs’ decision-making process and best practices among the NITAGs need to be developed. To meet these objectives, several initiatives need to be implemented:

- Terms of reference of all NITAGs should be well defined and should cover all current NETAGs’ practices.
- The decision framework should be transparent, structured, reproducible, and evidence based, and should follow a standardized process such as GRADE to provide reliable and robust assessments.
- Transparency of communication should be considered a key issue. Public access to agendas, recommendations, and technical reports should be standard. Ideally meetings should be open to the public.
- NITAGs’ assessment should include economic considerations (cost-effectiveness and budget impact analysis). However, this process should be kept separated from pricing and reimbursement pathways and overseen by another body.

*Walter Ricciardi* Professor European Public Health Association and Department of Public Health Catholic University of the Sacred Heart Rome, Italy *Mondher Toumi* Professor Faculty of Medicine – Public Health University Aix-Marseille Marseille, France

## References

[1] Wicker S, Maltezou HC (2014). Vaccine-preventable diseases in Europe: Where do we stand?. Expert Rev Vaccines.

[2] Ehreth J (2003). The global value of vaccination. Vaccine.

[3] Beutels P (2002). Economic evaluation of vaccination programmes in humans: A methodological exploration with application to hepatitis B, varicella-zoster, measles, pertussis, hepatitis A and pneumococcal vaccination.

[4] Antonova EN, Rycroft CE, Ambrose CS, Heikkinen T, Principi N (2012). Burden of paediatric influenza in Western Europe: A systematic review. BMC Public Health.

[5] Glaser K, Montserrat ER, Waginger U, Price D, Stuchbury R, Tinker A Grandparenting in Europe and the US.

[6] Dex S, Joshi H Millennium Cohort Study first survey: A user's guide to initial findings.

[7] Bonanni P, Boccalini S, Bechini A, Banz K (2008). Economic evaluation of varicella vaccination in Italian children and adolescents according to different intervention strategies: The burden of uncomplicated hospitalised cases. Vaccine.

[8] De Wals P, Blackburn M, Guay M, Bravo G, Blanchette D, Douville-Fradet M (2001). Burden of chickenpox on families: A study in Quebec. Can J Infect Dis.

[9] Coudeville L, Brunot A, Giaquinto C, Lucioni C, Dervaux B (2004). Varicella vaccination in Italy: An economic evaluation of different scenarios. Pharmacoeconomics.

[10] Banz K, Wagenpfeil S, Neiss A, Hammerschmidt T, Wutzler P (2004). The burden of varicella in Germany. Potential risks and economic impact. Eur J Health Econ.

[11] Van der Wielen M, Giaquinto C, Gothefors L, Huelsse C, Huet F, Littmann M (2010). Impact of community-acquired paediatric rotavirus gastroenteritis on family life: Data from the REVEAL study. BMC Fam Pract.

[12] Loeppke R, Nicholson S, Taitel M, Sweeney M, Haufle V, Kessler RC (2008). The impact of an integrated population health enhancement and disease management program on employee health risk, health conditions, and productivity. Popul Health Manag.

[13] OECD Health at a glance 2011: OECD indicators.

[14] World Health Organisation The global burden of disease: 2004 update.

[15] Barnighausen T, Bloom DE, Cafiero-Fonseca ET, O'Brien JC (2014). Valuing vaccination. Proc Natl Acad Sci USA.

[16] Soriano-Gabarro M, Mrukowicz J, Vesikari T, Verstraeten T (2006). Burden of rotavirus disease in European Union countries. Pediatr Infect Dis J.

[17] Harris JP, Jit M, Cooper D, Edmunds WJ (2007). Evaluating rotavirus vaccination in England and Wales. Part I. Estimating the burden of disease. Vaccine.

[18] Giaquinto C, Van Damme P, Huet F, Gothefors L, Van der Wielen M (2007). Costs of community-acquired pediatric rotavirus gastroenteritis in 7 European countries: The REVEAL study. J Infect Dis.

[19] Huet F, Largeron N, Trichard M, Miadi-Fargier H, Jasso-Mosqueda G (2007). Burden of paediatric rotavirus gastroenteritis and potential benefits of a universal rotavirus vaccination programme with RotaTeq in France. Vaccine.

[20] Public Health England Meningococcal disease: Guidance, data and analysis.

[21] Trotter CL, Edmunds WJ (2002). Modelling cost effectiveness of meningococcal serogroup C conjugate vaccination campaign in England and Wales. BMJ.

[22] Kaye P, Malkani R, Martin S, Slack M, Trotter C, Jit M Invasive pneumococcal disease (IPD) in England & Wales after 7-valent conjugate vaccine (PCV7): Potential impact of 10 and 13-valent vaccines.

[23] Gleizes O, Desselberger U, Tatochenko V, Rodrigo C, Salman N, Mezner Z (2006). Nosocomial rotavirus infection in European countries: A review of the epidemiology, severity and economic burden of hospital-acquired rotavirus disease. Pediatr Infect Dis J.

[24] van Seventer R, Sadosky A, Lucero M, Dukes E (2006). A cross-sectional survey of health state impairment and treatment patterns in patients with postherpetic neuralgia. Age Ageing.

[25] Charfi R, El Aidli S, Zaiem A, Kastalli S, Srairi S, Daghfous R (2012). Adverse drug reactions in older adults: A retrospective study from pharmacovigilance. Therapie.

[26] IARC European code against cancer.

[27] Blank PR, Schwenkglenks M, Sardos CS, Patris J, Szucs TD (2013). Population access to new vaccines in European countries. Vaccine.

[28] Ricciardi GW, Toumi M, Weil-Olivier C, Ruitenberg E, Dankó D, Duru G (2015). Comparison of NITAG policies and working processes in selected developed countries. Vaccine.

[29] Duclos P (2010). National Immunization Technical Advisory Groups (NITAGs): Guidance for their establishment and strengthening. Vaccine.

